# Up-regulated HMGB1 in EAM directly led to collagen deposition by a PKCβ/Erk1/2-dependent pathway: cardiac fibroblast/myofibroblast might be another source of HMGB1

**DOI:** 10.1111/jcmm.12324

**Published:** 2014-06-09

**Authors:** Zhaoliang Su, Jingping Yin, Ting Wang, Yingkun Sun, Ping Ni, Rui Ma, Haitao Zhu, Dong Zheng, Huiling Shen, Wenlin Xu, Huaxi Xu

**Affiliations:** a The Central Laboratory, The Fourth Affiliated Hospital of Jiangsu UniversityZhenjiang, China; b Department of Immunology & Laboratory Immunology, Jiangsu UniversityZhenjiang, China; c Department of Laboratory Medicine, The First Affiliated Hospital of Soochow UniversitySuzhou, China

**Keywords:** HMGB1, experimental autoimmune myocarditis, cardiac fibrosis

## Abstract

High mobility group box 1 (HMGB1), an important inflammatory mediator, is actively secreted by immune cells and some non-immune cells or passively released by necrotic cells. HMGB1 has been implicated in many inflammatory diseases. Our previous published data demonstrated that HMGB1 was up-regulated in heart tissue or serum in experimental autoimmune myocarditis (EAM); HMGB1 blockade could ameliorate cardiac fibrosis at the last stage of EAM. And yet, until now, no data directly showed that HMGB1 was associated with cardiac fibrosis. Therefore, the aims of the present work were to assess whether (1) up-regulated HMGB1 could directly lead to cardiac fibrosis in EAM; (2) cardiac fibroblast/myofibroblasts could secrete HMGB1 as another source of high-level HMGB1 in EAM; and (3) HMGB1 blockade could effectively prevent cardiac fibrosis at the last stage of EAM. Our results clearly demonstrated that HMGB1 could directly lead to cardiac collagen deposition, which was associated with PKCβ/Erk1/2 signalling pathway; furthermore, cardiac fibroblast/myofibroblasts could actively secrete HMGB1 under external stress; and HMGB1 secreted by cardiac fibroblasts/myofibroblasts led to cardiac fibrosis *via* PKCβ activation by autocrine means; HMGB1 blockade could efficiently ameliorate cardiac fibrosis in EAM mice.

## Introduction

Dilated cardiomyopathy (DCM) is one of the leading causes of severe heart failure in young patients and often evolves from viral myocarditis. Clinical data indicated that post-infectious autoimmune response promotes disease development [1]. Experimental autoimmune myocarditis (EAM) is a mouse model for CD4^+^ Th cell-mediated post-infectious myocarditis, characterized by inflammatory cells' infiltration of the myocardium, cardiomyocytes necrosis and cardiac fibroblasts/ myofibroblasts collagen deposition [2] and can be induced by inoculation with coxsackie B virus or cardiac myosin heavy chain (MyHC)-α_614-629_ peptide in susceptible mouse strains [3,4]. Our previous published data demonstrated that high mobility group box 1 (HMGB1) was up-regulated in heart tissue or serum in EAM model induced by MyHC-α_614-629_; and that the high-level HMGB1 contributed to the pathogenesis of EAM by promoting Th17 cells expansion [5].

HMGB1, a chromatin-associated non-histone nuclear protein and extracellular damage-associated molecular patterns (DAMPs), is a critical regulator of cell death and survival [6]. HMGB1 can be actively secreted by immune cells' exposure to pathogen-associated molecular patterns (PAMPs), DAMPs or under external stress such as tumour necrosis factor (TNF)-α and interleukin (IL)-1 [7] or passively released by necrotic cells or apoptotic cells. Recently, more and more data indicated that some non-immune cells, such as, endothelial cell, hepatocytes [8], pituicytes [9], cardiomyocytes [10] and enterocytes [11] also can actively secrete HMGB1.

HMGB1 secreted into extracellular milieu, acting as an alarmin or molecular chaperones, interacts with endogenous receptor for advanced glycation end products (RAGE) [12], or exogenous toll-like receptor 2/4/9 (TLR2/4/9) [13,14] and CD24/Siglec-10 [15], and induces the expression of pro-inflammatory cytokines, chemokines and adhesion molecules [16,17]. Initial studies demonstrated that HMGB1 was a late mediator of sepsis; however, the recent publications and our data indicated that HMGB1 was associated with CD4^+^ T helper cell development [5,18], autoimmune diseases [19], cancer [20–22], trauma, ischaemia-reperfusion injury [23,24], tissue repair and regeneration [25,26] and cardiovascular diseases [27]. In addition, there are also some data which indicated that HMGB1 played critical roles in pulmonary fibrosis [28], cystic fibrosis [29] and renal fibrosis [30]. However, our published data also demonstrated that HMGB1 blockade could ameliorate cardiac pathology and cardiac fibrosis at the last-stage of EAM [5]. And yet, until now, no data directly showed that HMGB1 was associated with cardiac fibrosis or whether cardiac fibroblasts/myofibroblasts could actively secrete HMGB1 under external stress.

Although our published data demonstrated that HMGB1 was up-regulated in heart tissue and serum; furthermore, HMGB1 blockade could ameliorate cardiac fibrosis in EAM; however, whether high-level HMGB1 in EAM could directly lead to cardiac fibrosis and what were the sources of high-level HMGB1 remained to be investigated. However, some publishing showed that cardiac endothelial cell and cardiomyocytes [10] could actively secrete HMGB1 and whether cardiac fibroblasts/myofibroblasts, as another important component of heart, also could actively secrete HMGB1. Therefore, the aims of the present work were to assess whether (1) high-level HMGB1 could directly lead to cardiac fibrosis in EAM; (2) cardiac fibroblasts/myofibroblasts could actively secrete HMGB1 as another source of high-level HMGB1 in EAM; and (3) HMGB1 blockade could effectively prevent cardiac fibrosis at the last stage of EAM. Our results clearly demonstrated that HMGB1 could directly lead to cardiac collagen deposition, which was associated with PKCβ/Erk1/2 signalling pathway; furthermore, cardiac fibroblasts/myofibroblasts could actively secrete HMGB1; and HMGB1, actively secreted by cardiac fibroblasts/myofibroblasts led to cardiac fibrosis *via* PKCβ activation by autocrine means; HMGB1 blockade could efficiently ameliorate cardiac fibrosis in EAM mice.

## Materials and methods

### Mice

BALB/c mice, 6–8 weeks old, were purchased from the Animal Center of Yangzhou University and maintained in the Animal Center of Jiangsu University. All animal procedures in the present study were in compliance with the Guide for the Care and Use of Laboratory Animals (NIH Publication No. 85-23, revised 1996). The experimental protocols were approved by our institutional ethics committee. Tissue for primary cell cultures and assays was isolated from mice after killing with an overdose of pentobarbital sodium (about 30 mg/kg body weight, i.p.) and confirmation of death by either cervical dislocation.

### Cardiac fibroblasts isolation, culture and treatment

Cardiac fibroblasts were isolated from 1- to 2-week-old BALB/c mice according to previous reports [31,32]. Briefly, hearts were removed under sterile conditions, cut into pieces and digested with 0.1% trypsin/0.2% type II collagenase (Invitrogen Life Technologies, Shanghai, China) at 37°C for 1 hr, until the tissue blocks disappeared. Dissociated cells were then centrifuged at 350 g, resuspended in DMEM with 10% foetal bovine serum (FBS), 2 mM glutamine, 100 μg/ml streptomycin and 100 U/ml penicillin; after 2 hrs, non-adherent cells were removed and adherent cells were maintained in 5% CO_2_ at 37°C, and the medium was replaced every 2–3 days. When the cells approached confluence, they were passage after 1:3 dilutions with fresh medium. Cardiac fibroblasts/myofibroblasts from passages 3–5 were used in every experiment. The cells were morphologically homogeneous with typical bipolar configuration observed by inverse microscopy.

Before challenge by LPS of *Escherichia coli* serotype 055:B5 (Sigma-Aldrich, Shanghai, China), the viability of cardiac fibroblasts/myofibroblasts was assessed by trypan blue staining. Briefly, we placed 0.5 ml cells suspension (dilute cells in complete medium without serum to an approximate concentration of 1–2 × 10^5^ cells per ml) in a screw cap tube, added 0.1 ml of 0.4% trypan blue staining and then mixed and incubated for 5 min. at room temperature, then filled a haemocytometer for cell counting by microscope [33].

### RT-qPCR Assay

TLR2, TLR4, TLR9, RAGE, HMGB1, Collagen type I/III (Col1/3) and Osteopontin (OPN) message levels were assessed by RT-qPCR according to the method previously described. Briefly, total RNA was isolated from cardiac fibroblasts/myofibroblasts by using TRIzol reagent (Invitrogen Life Technologies) according to the manufacturer's protocol and reverse transcribed into first-strand cDNA by use of the moloney murine leukaemia virus reverse transcriptase system. After cDNA synthesis, real-time PCR was performed with SYBR Green Supermix (TransGen Biotech, Beijing, China) by using Rotor-Gene (RG)-6000 (Corbett Research, Mortlake, Australia) with β-actin as an internal control. Quantification of gene expression was calculated relative to β-actin. All the primers were listed in Table 1.

**Table 1:** Primers sequences used in the present study

Genes	Sequence	Genes	Sequence
TLR2	cagacgtagtgagcgagctg	Col1a1	ttgcttcccagatgtcctatg
	ggcatcggatgaaaagtgtt		cttccccatcatctccattct
TLR4	ttcacctctgccttcactaca	Col1a3	caatatgcccacagccttct
	gggacttctcaaccttctcaa		ggccaccagttggacatgat
TLR9	gaaagcatcaaccacaccaa	OPN	tgaagagcggtgagtctaagg
	acaagtccacaaagcgaagg		tggaatgctcaagtctgtgtg
RAGE	gggtgctggttcttgctc	TIMP1	cagaaccgcagtgaagag
	ccctcgcctgttagttgc		ggatagataaacagggaaaca
HMGB1	ggctgacaaggctcgttatg	MMP2	ccccgatgctgatactga
	gggcggtactcagaacagaac		tgtccgccaaataaacc
MMP1a	tgaatggcaaggagatg	β-actin	tggaatcctgtggcatccagaaac
	aacgaggattgttgtgagta		taaaacgcagctcagtaacagtccg

### Immunofluorescence staining

Immunofluorescence staining of cardiac fibroblasts/myofibroblasts was performed as described previously [34]. Briefly, after challenge by LPS (500 ng/ml), medium was removed from the plates and cells were washed twice with PBS. Cardiac fibroblasts/myofibroblasts were fixed with 4% paraformaldehyde solubilized in PBS/0.1% Triton-X100 for 30 min. at room temperature, then blocked for 1 hr with 1% BSA. The primary anti-HMGB1 antibody (ab18256; Abcam, Shanghai, China) was applied for 2 hrs at room temperature. After washing, secondary antibody labelled by PE was added for 1.5 hrs. Finally, cells were stained with Hoechst 33342 (Sigma-Aldrich) for 5 min. Sections were viewed with fluorescence microscope (Olympus, Beijing, China).

### Western blot analysis

Cardiac fibroblasts/myofibroblasts were lysed with RIPA buffer and proteinase inhibitor mixture (PMSF). Nuclear and cytoplasm protein were separated by using nuclear and cytoplasm protein extraction kit (78833; Thermo Pierce, Rockford, IL, USA), according to manufacturer's instructions. Protein concentration was assessed by Bradford assay (Bio-Rad Laboratories, Shanghai, China). Total proteins were electrophoresed on 12% SDS-PAGE gels and transferred onto polyscreen PVDF transfer membranes (PerkinElmer, Boston, MA, USA). Membranes were blocked with 5% (w/v) non-fat milk in tris-buffered saline (TBS) containing 0.1% Tween 20 for 1 hr at room temperature and incubated overnight with primary anti-col3A1, anti-col1A1, anti-β-actin (sc-28888, sc-8784, sc-47778, respectively, Santa Cruz biotechnology Inc, Santa Cruz, CA, USA), anti-OPN (ab8448; Abcam) at 4°C, respectively. After washing, HRP-conjugated secondary antibody was added for 1 hr at 37°C. Detection was performed with enhanced chemiluminescence (ECL) and relevant blots were quantified by densitometry by using the accompanying computerized image analysis program.

### siRNA experiment in cardiac fibroblasts/myofibroblasts

Control siRNA and HMGB1 siRNA were obtained from Guangzhou RiboBio Co., Ltd, Guangzhou, China. Control siRNA (sin05815122147) consists of a scrambled sequence that will not lead to the specific degradation of any known cellular mRNA. HMGB1 siRNA is a pool of three target-specific 19- to 25-nt siRNAs (sib0941383447, sib11129130839 and sib11129130857, respectively) designed to knock down gene expression. siRNA was prepared according to the transfection protocol for cell cultures. Briefly, siRNA transfection reagent Lipofectamine 2000 (11668-019; Invitrogen Life Technologies) mixture of 1 ml was co-incubated with cardiac fibroblasts/myofibroblasts for 6 hrs in a 5% CO_2_ incubator at 37°C, and then the same amount of DMEM 20% FBS was added. An additional incubation was performed for 18 hrs, and then the procedure for conditioned media was carried out.

### Induction of experimental autoimmune myocarditis

Mice EAM models were induced following the protocol of our laboratory [5]. Briefly, mice were inoculated with 100 μg of MyHC-α (MyHC-α_614–629_; Ac-SLKLMATLFSTYASAD-OH), emulsified at 1:1 ratio in PBS/CFA at days 0 and 7. On day 54, the mice were anaesthetized with pentobarbital sodium (30 mg/g body weight, i.p.) and killed by cervical dislocation. Heart tissues were collected for analysis.

### Histopathology

Mice hearts were fixed in 10% formalin, paraffin-embedded, and stained with haematoxylin and eosin. Cardiac fibrosis was evaluated by sirius-red staining [35]. Briefly, mice hearts were fixed in 4% paraformaldehyde, paraffin-embedded, dewaxed and put into blue lapis lazuli liquid for 7 min., and then the slides were washed in double-distilled H_2_O for three times. Samples were kept in saturated picro-sirius red liquid for 30 min. and dehydrated with ethanol.

### Migration experiments

Migration assays were performed by using 6-well Transwell plates with an 8-μm–pore-size polycarbonate filter (Costar, Cambridge, MA, USA), as previously described [36]. Briefly, cardiac fibroblasts/myofibroblasts were placed in the upper chamber and grown in complete medium. In the lower chamber with or without HMGB1, transwell plates were then incubated for 6 hrs at 37°C in a 5% CO_2_ humidified atmosphere.

### MTT assay

Cardiac fibroblast/myofibroblast proliferation was evaluated by MTT assay. Briefly, 5 × 10^4^ cells/well/200 μl were cultured with 50, 100, 500 ng/ml HMGB1 or without HMGB1 for 24 hrs in a 96-well flat-bottom plate. After incubation, the plate was centrifuged for 5 min. at 800 × *g* at 4°C. The supernatants (150 μl/well) were removed and 50 μl of fresh medium and 25 μl of MTT solution were added in each well and the plate was incubated for 4 hrs. After addition of 100 μl of stop solution in each well, the plate was incubated overnight in dark at 37°C and the absorbance was measured at 540 nm by using Bio-Rad Automated EIA Analyzer (Bio-Rad).

### Statistical analysis

All statistical analyses were performed by using Prism 5 (Graph Pad Software, La Jolla, CA, USA). Data were expressed as the mean ± SD. Comparisons between groups were performed by using the paired *t*-test or one-way anova with Bonferroni correction. A *p* value of <0.05 was considered statistically significant difference.

## Results

### HMGB1 could directly lead to cardiac collagen deposition of cardiac fibroblasts/myofibroblasts by a PKCβ/Erk1/2-dependent signalling pathway

**Fig. 1:**
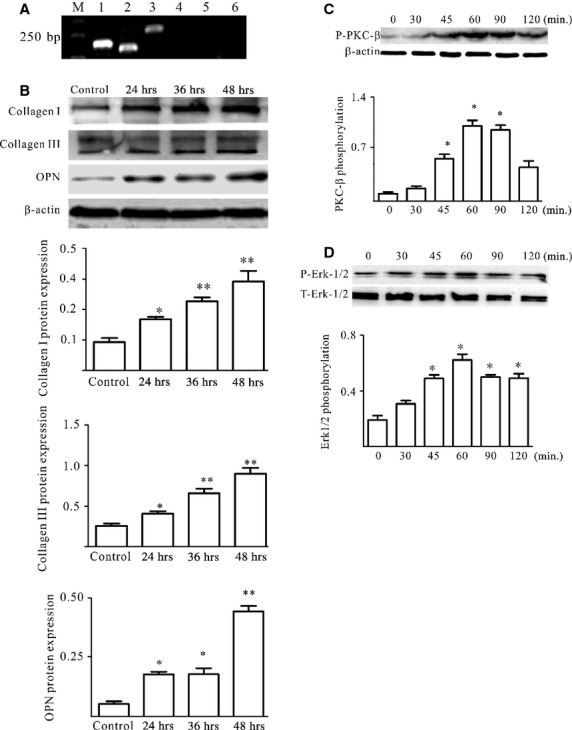
HMGB1 could directly lead to cardiac collagen deposition of cardiac fibroblasts/myofibroblasts. (**A**) TLR2 and TRL4 were expressed on cardiac fibroblasts/myofibroblasts. M,1-6 indicated DL2000 marker, β-actin, TLR2, TRL4, TLR9, RAGE and blank, respectively. (**B**) The levels of Col1, Col3 and OPN were detected by western blot in cardiac fibroblasts/myofibroblast lysates treated with 100 ng/ml HMGB1 for 24, 36 and 48 hrs. (**C** and **D**) HMGB1 activated the PKCβ/Erk1/2 in cardiac fibroblast/myofibroblast. Cardiac fibroblasts/myofibroblasts were treated by 100 ng/ml HMGB1. At the points indicated, cardiac fibroblasts/myofibroblasts were harvested; phosphorylated PKC β (p-PKC β; anti-phosphorylated PKCβII, 2252-1; Epitomics, Burlingame, CA, USA) and Erk1/2 (9101S; Cell Signaling Technology, Boston, MA, USA) levels were assessed by western blot. Representative blots are shown above and densitometric analyses, below. All data were obtained from three independent experiments.

Our published data demonstrated that HMGB1 was up-regulated in heart tissue and serum in EAM; furthermore, HMGB1 blockade could ameliorate cardiac fibrosis [5]; To address whether HMGB1 could directly lead to cardiac collagen deposition in EAM, cardiac fibroblasts were isolated and the purity was up to 95% after 3–5 passages (data not shown). And the candidate's receptors of HMGB1, for example TLR2, TLR4, TLR9 and RAGE, were detected. As shown in Figure 1A, TLR2 and TLR4 were expressed on cardiac fibroblasts/myofibroblasts. The primary cardiac fibroblasts/myofibroblasts were then treated by 100 ng/ml exogenous HMGB1 (H4652, Sigma-Aldrich) for 24, 36 and 48 hrs *in vitro*. The results showed that Col1, Col3 and OPN expression significantly increased after 24 hrs (Fig. 1B). Challenge of cardiac fibroblasts/myofibroblasts with HMGB1 resulted in a transient increase in phosphorylation of PKC-β and Erk1/2 within cardiac fibroblasts/myofibroblasts, peaking at 45–90 min., 45–120 min. and the phosphorylation level keeping up to 12 hrs, 4 hrs after challenge, respectively (Fig. 1C and D, Fig. S1). Taken together, these results clearly demonstrated that HMGB1 directly led to cardiac collagen deposition, which was associated with PKCβ/Erk1/2 signalling pathway.

**Fig. 2:**
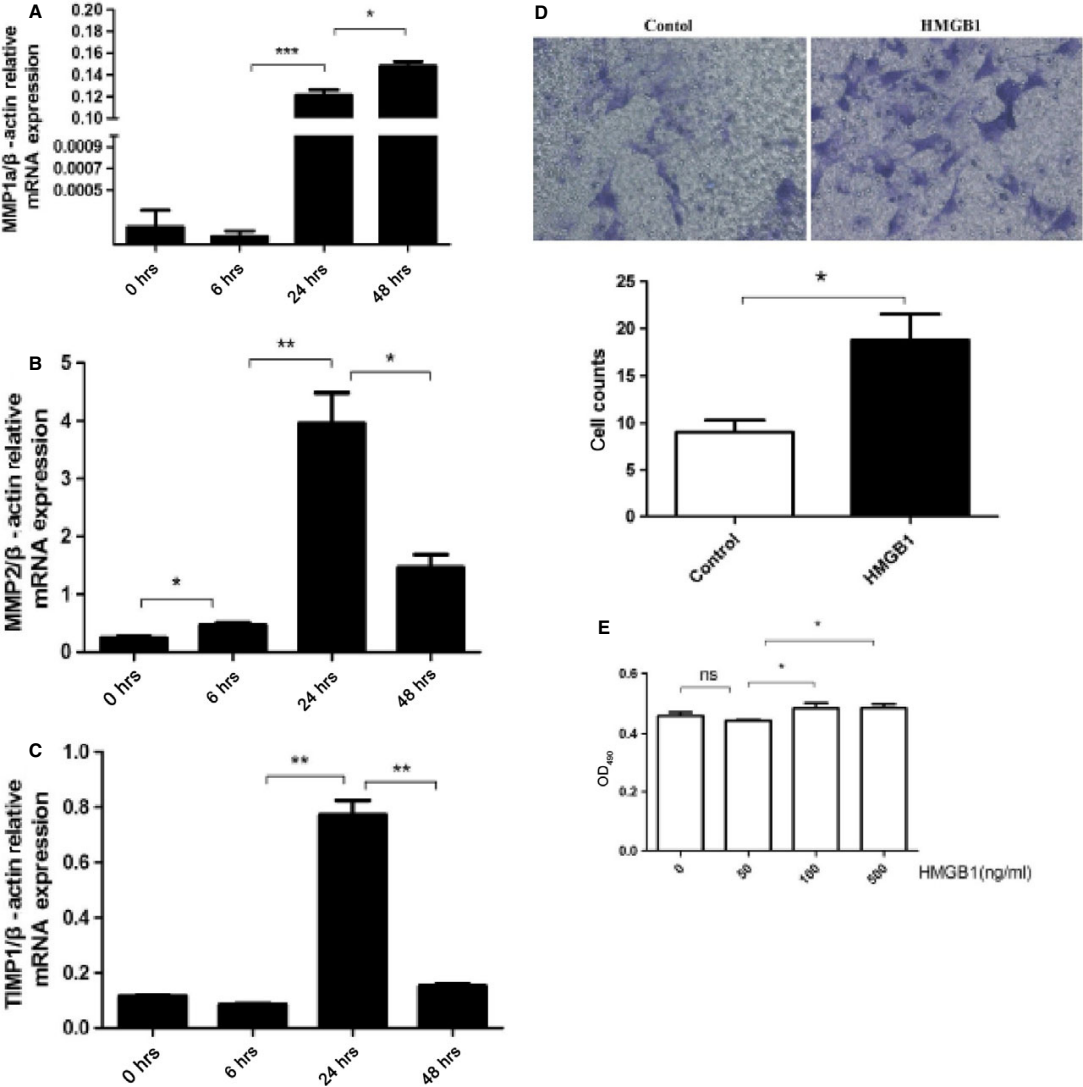
Cardiac fibroblasts/myofibroblasts were proliferation, migration, and expression MMP and TIMP after HMGB1 stimulus. (**A**–**C**) Cardiac fibroblasts/myofibroblasts were treated by 50 ng/ml HMGB1 for 6, 24 and 48 hrs. Cardiac fibroblasts/myofibroblasts were harvested at indicated point for assessment of MMP1/2 and TIMP1 mRNA by RT-qPCR. (**D**) Cell migration was performed by using 6-well Transwell plates with an 8-μm–pore-size polycarbonate filter, 1 × 10^4^ cardiac fibroblasts/myofibroblasts were placed in the upper chamber and grown in complete medium. In the lower chamber with or without HMGB1, transwell plates were then incubated for 6 hrs at 37°C in a 5% CO_2_ humidified atmosphere. The lower filter surface were fixed with ethanol and stained with Giemsa. The number of migrating cells was determined by counting cells in 10 random fields at ×200 magnification. (**E**) Cardiac fibroblasts/myofibroblasts were treated in 96-well plates and grown until 70–80% confluent, and then added 50, 100, 500 ng/ml HMGB1 to the culture medium. After 24 hrs, MTT was added to each well under sterile conditions, and the plates were incubated for 4 hrs at 37°C. Untransformed MTT was removed by aspiration, and formazan crystals were dissolved in dimethyl sulfoxide (150 μl/well). Formazan was quantified spectroscopically at 540 nm by using Bio-Rad Automated EIA Analyzer (Bio-Rad). All the experiments were performed in triplicate with different preparations of cardiac fibroblasts/myofibroblasts. *P* < 0.05 was considered statistically significant.

Furthermore, fibroblasts/myofibroblasts are not only the main producers of collagens but also the major source of matrix metalloproteinases (MMPs) and their tissue inhibitors (TIMPs), which initiate pathological remodelling by altering ECM turnover [37]. Therefore, MMP1/2 and TIMP1 in cardiac fibroblasts/myofibroblasts were detected after HMGB1 treatment. Furthermore, cardiac fibroblasts/myofibroblasts proliferation and migration were also detected after HMGB1 treatment. The results showed that HMGB1 not only up-regulated the MMP1/2 and TIMP1 expression in cardiac fibroblasts/myofibroblasts but also promoted cardiac fibroblasts/myofibroblasts proliferation and migration (Fig. 2).

### Cardiac collagens deposition accompanying HMGB1 up-regulation and HMGB1 blockade ameliorated cardiac fibrosis in EAM mice

**Fig. 3:**
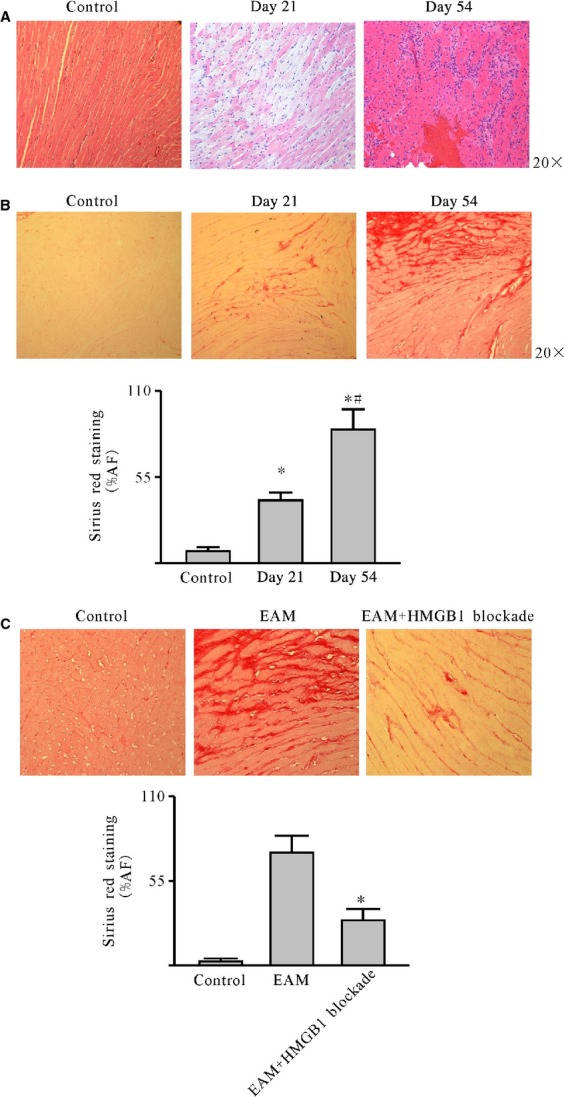
HMGB1 blockade ameliorated cardiac fibrosis in EAM mice. After the model induction, the mice were killed on day 21 and day 54 respectively. The cardiac sections were stained with Sirius Red for collagen deposition and haematoxylin and eosin. (**A**) Haematoxylin and eosin-stained sections (20×). (**B**) Collagen was deposited in EAM mice heart accompanying the HMGB1 up-regulation (20×). (**C**) HMGB1 blockade decreased collagen deposition and ameliorated fibrosis (40×). A representative image is shown in the upper panel for each group. The fraction of positively stained areas is presented as mean ± SD with five mice for each group. *P < 0.05.

*In vivo*, on day 21 after the model induction, the cardiomyocytes began swelling, lysis and eventually necrosis replacing by proliferated cardiac fibroblasts/myofibroblasts accompany HMGB1 up-regulation (Fig. 3A); this observation was further confirmed through collagen staining by sirius red. The percentage of sirius red-stained area significantly increased in EAM mice on day 54 [8 ± 6.5% (control) *versus* 86 ± 19% (day 54); Fig. 3B].

To address whether HMGB1 directly led to collagen deposition in EAM mice heart, two lines of hybridoma (1D2F4E3 and 2D4E3A2) generating mAbs specific for the HMGB1 B box domain were employed to block HMGB1 [5,38]. After blockade at the last stage of EAM, as the Figure 3C shows, the percentage of sirius red-stained area markedly reduced [82 ± 17% (day 54) *versus* 35 ± 4.5% (after blockade)]. Taken together, these results clearly demonstrate that HMGB1 blockade ameliorated cardiac fibrosis in EAM.

### External stress could up-regulate HMGB1 expression of cardiac fibroblasts/myofibroblasts

**Fig. 4:**
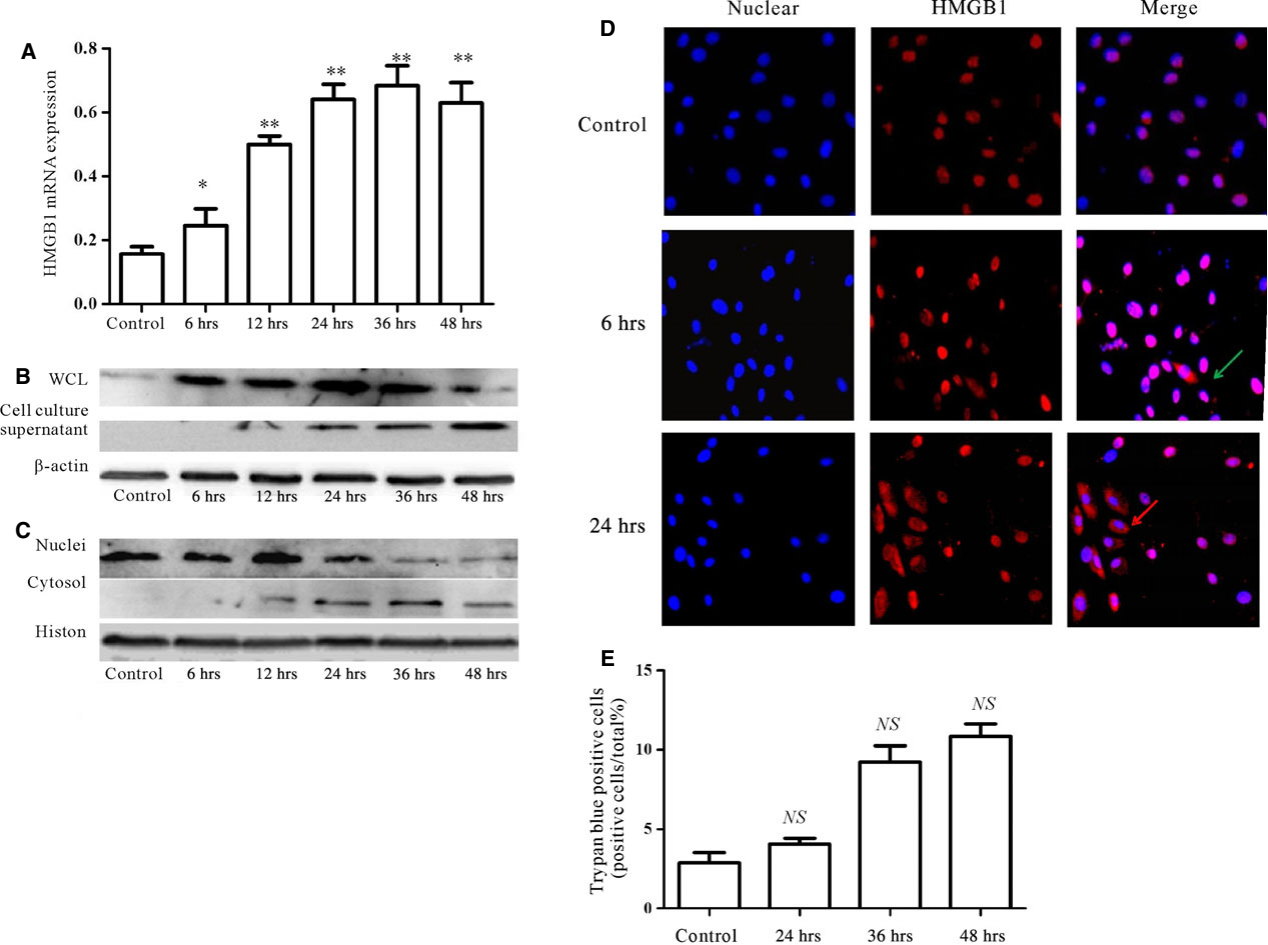
Cardiac fibroblasts/myofibroblasts could secrete HMGB1 under external stress. (**A**) Primary cardiac fibroblasts/myofibroblasts were challenged by 500 ng/ml LPS for 6, 12, 24, 36 and 48 hrs. Cardiac fibroblasts/myofibroblasts were harvested at times indicated. HMGB1 mRNA was detected by RT-qPCR. After 6 hrs, the HMGB1 mRNA significantly increased. Values were expressed as HMGB1 compared with β-actin. Data were means ± SD from three independent experiments. (**B** and **C**) HMGB1 protein expression. Cardiac fibroblasts/myofibroblasts were challenged by 500 ng/ml LPS for 6, 12, 24, 36 and 48 hrs. Cardiac fibroblasts/myofibroblasts and their supernatants were harvested at the indicated point. Whole cell lysates (WCL), nuclei and cytosol proteins were extracted. The levels of proteins were assessed by western blot. β-actin was used as a loading control for WCL and cytosol; histon was as a nuclei protein control. (**D**) HMGB1 was shuttled from nucleus to cytoplasm in cardiac fibroblasts/myofibroblasts under external stress. After 6 hrs, challenged by 500 ng/ml LPS, HMGB1 began to shuttle. The arrows showed the HMGB1 expression in the cytoplasm. (**E**) Cardiac fibroblasts/myofibroblasts were challenged by 500 ng/ml LPS for 24, 36 and 48 hrs and then stained with Trypan blue. Data were obtained from three independent experiments. *P*-values were calculated by using the paired *t*-test; *P* < 0.05 was considered statistically significant. **P < 0.01 compared with control. *NS* means No statistical significance.

As our published data demonstrated that HMGB1 was up-regulated in heart tissue and serum [5], we also could speculate that high-level HMGB1 originated from necrosis cells, infiltrated immune cells, cardiac endothelial cell and cardiomyocytes [10]. We also want to find out whether cardiac fibroblasts/myofibroblasts, as another critical component of heart, also could secrete HMGB1 under external stress. The primary cardiac fibroblasts were challenged by 500 ng/ml LPS, a classical method to explore HMGB1 secretion by different cells. As shown in Figure 4A and B, after 6 hrs, HMGB1 mRNA and proteins were obviously increased; similar data were also obtained in the cell culture supernatants (Fig. 4B). To further confirm the HMGB1 shuttle, the nuclei and cytosol proteins were extracted; as shown in Figure 4C, after 6 hrs, HMGB1 began to shuttle from nuclear to cytoplasm. And these data were further confirmed by immunofluorescence staining (Fig. 4D).

As shown in Figure 4E, primary cardiac fibroblast/myofibroblast viability was not affected by 500 ng/ml LPS challenge for 48 hrs. These findings clearly demonstrated that cardiac fibroblast/myofibroblast could secrete HMGB1 under external stress.

### HMGB1, secreted by cardiac fibroblast/myofibroblast under external stress, up-regulated Col1, Col3 and OPN expression *via* PKC β activation by autocrine means

**Fig. 5:**
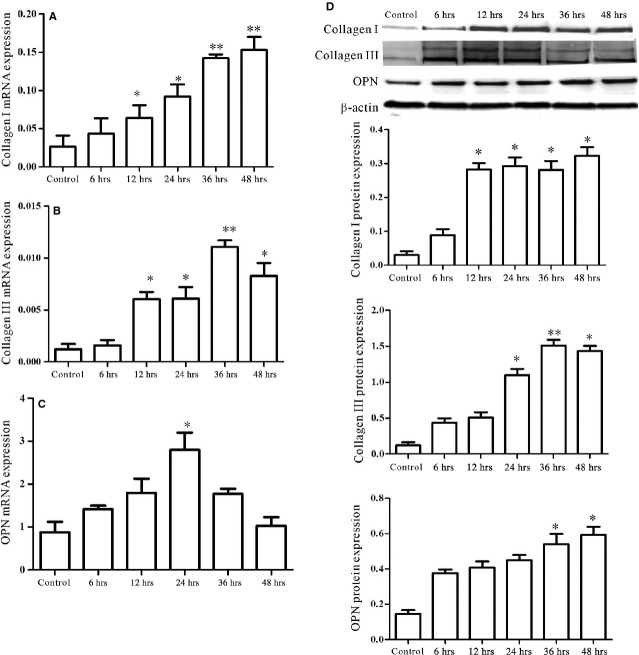
Accompanying HMGB1 secretion, Col1, Col3 and OPN expression were increased in cardiac fibroblasts/myofibroblasts. Cardiac fibroblasts/myofibroblasts were challenged by 500 ng/ml LPS for 6, 12, 24, 36 and 48 hrs. (**A**–**C**) Cardiac fibroblasts/myofibroblasts were harvested at indicated point for assessment of Col1, Col3 and OPN mRNA by RT-qPCR. After 12, 12 and 24 hrs, the Col1, Col3 and OPN mRNA significantly increased, respectively. Values were expressed as Col1, Col3 and OPN compared with β-actin, respectively. (**D**) Western blot analysis of Col1, Col3 and OPN protein levels in cardiac fibroblasts/myofibroblast lysates (upper panel). Densitometric analysis blots are shown below. β-actin served as a loading control. All data were given as means ± SD from three independent experiments. All values were tested by paired *t*-test. *P* < 0.05 was considered statistically significant. **P < 0.01 compared with control.

As external stress could up-regulate HMGB1 expression of cardiac fibroblasts/myofibroblasts, the next question to examine was whether up-regulated HMGB1 could lead to collagen deposition of cardiac fibroblasts/myofibroblasts by autocrine means. Our results showed that, accompanying HMGB1 secretion, Col1, Col3 and OPN mRNA of cardiac fibroblasts/myofibroblasts were significantly increased at 12, 12 and 24 hrs respectively (Fig. 5A–C). In line with mRNA expression, elevated levels of Col1, Col3 and OPN protein were observed in the challenged cardiac fibroblasts/myofibroblasts (Fig. 5D).

**Fig. 6:**
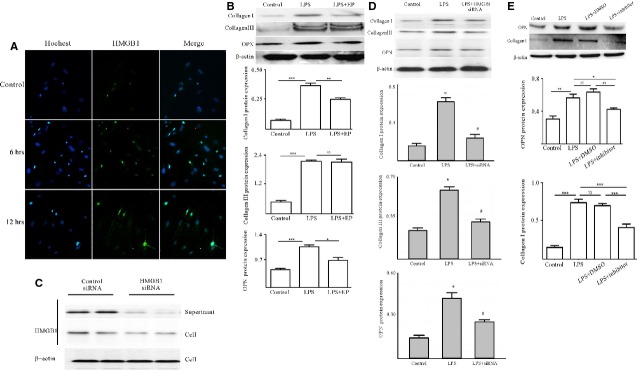
HMGB1, secreted by cardiac fibroblasts/myofibroblasts under external stress, up-regulated the expression of Col1, Col3 and OPN *via* PKC β activation by autocrine means. (**A**) HMGB1 expression was limited to nucleus in cardiac fibroblasts/myofibroblasts under external stress with EP existence for 6 or 12 hrs. (**B**) Col1, Col3 and OPN expression down-regulated on cardiac fibroblasts/myofibroblasts because of HMGB1 secretion blockade by EP. (**C**) HMGB1 siRNA successfully inhibited HMGB1 synthesis of cardiac fibroblasts/myofibroblasts under external stress. Reduction of HMGB1 release in cell culture supernatant was accompanied by HMGB1 down-regulation in cell lysates. (**D**) Col1, Col3 and OPN expression down-regulated on cardiac fibroblasts/myofibroblasts because of HMGB1 synthesis inhibition by HMGB1 siRNA. (**E**) HMGB1, secreted by cardiac fibroblast/myofibroblast under external stress, up-regulation the Col1, Col3 and OPN expression was abrogated by PKC β inhibitor. Cardiac fibroblasts/myofibroblasts were pre-treated with 10 μM PKCβ inhibitor, and then exposed to 500 ng/ml LPS for 24 hrs. Western blot analysed Col1 and OPN expression in cardiac fibroblast/myofibroblast lysates. Representative blots are shown above and densitometric analysis shown below. β-actin was as a loading control. Data were means ± SD from three independent experiments. All values were tested by paired *t*-test. *P* < 0.05 was considered statistically significant. **P < 0.01 compared with control.

To address whether the effect of cardiac collagen deposition was because of HMGB1 secretion by cardiac fibroblast/myofibroblast under external stress *via* autocrine means, two additional experiments were performed, one adding HMGB1 secretion inhibitor ethyl pyruvate (EP) [39,40] and the other suppressing endogenous HMGB1 synthesis by siRNA. When EP was added, the HMGB1 expression was limited to nucleus of cardiac fibroblasts/myofibroblasts (Fig. 6A); Col1 and OPN expression were obviously decreased; although Col3 expression was also decreased, there was no statistically significant difference (Fig. 6B). Furthermore, when endogenous HMGB1 expression in cardiac fibroblasts/myofibroblasts were inhibited by siRNA (Fig. 6C and D), Col1, Col3 and OPN expression increase was blocked.

To further confirm HMGB1, secreted by cardiac fibroblasts/myofibroblasts under external stress, to up-regulate Col1, Col3 and OPN expression *via* PKC β activation through autocrine, the PKC β inhibitor was also employed to block HMGB1 secretion. First, cardiac fibroblasts/myofibroblasts were exposed to 10 μM PKC β inhibitor for 2 hrs, followed by treatment with LPS for 24 hrs. As Figure 6E shows, Col1 and OPN expression was obviously decreased. These results clearly demonstrated that HMGB1, secreted by cardiac fibroblasts/myofibroblasts under external stress, contributed to cardiac collagen deposition *via* PKC β activation through autocrine.

## Discussion

During exploration of aetiology and pathophysiology for inflammatory diseases, many molecules have been identified as endogenous damage-associated molecules with pro-inflammatory functions, which may be responsible for the sterile inflammation [41,42]. HMGB1 was among the most extensively studied. HMGB1 was actively secreted by immune cells and non-immune cells, such as, hepatocytes [8], pituicytes [9], cardiomyocytes [10] and enterocytes [11] or passive release from necrotic cells. HMGB1 secreted into extracellular milieu is involved in many inflammatory, autoimmune diseases and cardiovascular disease by binding with its receptors [27]. Our published data demonstrated that HMGB1 was up-regulated in heart tissue or serum in EAM; HMGB1 blockade could ameliorate cardiac fibrosis at the last stage of EAM [5]. However, it is reported that HMGB1 plays an important role in many tissues fibrosis, such as pulmonary fibrosis, cystic fibrosis, renal fibrosis and so on [28–30]. However, no report indicated whether HMGB1 was directly involved in cardiac fibrosis. Therefore, in the present work, we attempted to explore (1) whether high-level HMGB1 in EAM was a direct risk factor of myocardial fibrosis; (2) except for immune cells, endothelial cells, cardiomyocytes and necrosis cells, whether cardiac fibroblasts/myofibroblasts, as another important component of heart, were also the critical sources of high-level HMGB1 in EAM.

Our results clearly demonstrated that HMGB1 directly led to cardiac collagen deposition by PKCβ/Erk1/2-dependent signalling pathway (Fig. 1). Of course, the PKCβ or Erk1/2 deficiency mice were needed to further confirm the conclusion. The other report also showed that HMGB1 was involved in the cardiac fibrosis by activation of TLRs downstream MyD88-dependent signalling pathway [43]. However, at the least, our data clearly demonstrated that HMGB1 was a direct risk factor of cardiac fibrosis; some reports were not consistent with our data; their data showed that exogenous HMGB1 could attenuates cardiac remodelling, and improves cardiac function after myocardial infarction (MI) by inducing myocardial regeneration [44,45]. We speculated that the reason that led to different conclusions, on the one hand, was the use of different models, the pathogenesis of which was not the same; on the other hand, the different sources of HMGB1 having different biological activity. To find out whether high-level HMGB1 had another sources in EAM, the cardiac fibroblasts were isolated and challenged by LPS, a classical method to explore HMGB1 secretion by non-immune cells because of its clear signal pathway. Furthermore, we clearly demonstrated that the cardiac fibroblasts/myofibroblast expressed the receptor TLR4 of LPS; however, we cannot make sure that MyHC-α_614-629_ can be recognized by the cardiac fibroblasts/myofibroblasts. Our results showed that HMGB was shuttled from nucleus to cytoplasm in cardiac fibroblasts/myofibroblasts under external stress (Fig. 4). In the next step, we should confirm whether the signal pathway of HMGB1 secretion by cardiac fibroblast/myofibroblast was consistent with macrophages. In addition, our data indicated that HMGB1, secreted by cardiac fibroblasts/myofibroblasts under external stress, contributed to the cardiac collagen deposition *via* PKC β activation through autocrine (Figs 5 6). To find out if the up-regulated HMGB1 could lead to cardiac collagen deposition *in vitro*, it was investigated whether HMGB1 blockade could ameliorate cardiac fibrosis at the last stage of EAM. Therefore, the mAb against HMGB1 B box was produced by a hybridoma technique. HMGB1 blockade significantly abated cardiac fibrosis at the last stage (Fig. 3). In terms of the contribution of HMGB1 to the development and progression of tissue fibrosis, conflicting results have been documented in different models, such as RAGE-deficient mice spontaneously developing lung fibrosis; furthermore, these mice showed enhanced resistance to bleomycin-induced lung fibrosis [46]. Nevertheless, in the present study, we could observe that HMGB1 blockade has a protective function in cardiac fibrosis in EAM mice.

## Conclusions

**Fig. 7:**
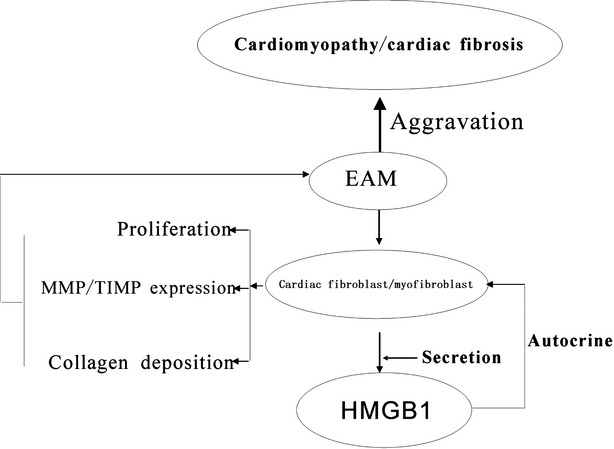
HMGB1 secreted by cardiac fibroblasts/myofibroblast under external stress contributed to collagen synthesis through autocrine pathway.

In conclusion, our previous work clearly demonstrated that HMGB1 was up-regulated in heart tissue or serum in EAM; HMGB1 blockade ameliorated cardiac fibrosis at the last stage of EAM. The present works further confirms that HMGB1 could directly lead to cardiac collagen deposition by a PKCβ/Erk1/2-dependent signalling pathway; HMGB1, secreted by cardiac fibroblasts/myofibroblasts under external stress, led to cardiac fibrosis *via* PKC β activation through autocrine; HMGB1 blockade could efficiently ameliorate cardiac fibrosis in EAM mice (Fig. 7).

## Supporting information

**Figure S1** HMGB1 activated the PKCβ/Erk1/2 in cardiac fibroblasts/myofibroblasts. Cardiac fibroblasts/myofibroblasts were treated by 100 ng/ml HMGB1. After 4, 8 and 12 hrs, cardiac fibroblasts/myofibroblasts were harvested; phosphorylated PKC β (p-PKC β) and phosphorylated Erk1/2 levels were assessed by western blot. Representative blots are shown above. Data were obtained from three independent experiments.
