# Supplementary figures and images for: Up-regulated HMGB1 in EAM directly led to collagen deposition by a PKCβ/Erk1/2-dependent pathway: cardiac fibroblast/myofibroblast might be another source of HMGB1
Source: J Cell Mol Med. 2014 Jun 9;18(9):1740–51. doi: 10.1111/jcmm.12324 (PMC4196650; doi:10.1111/jcmm.12324)

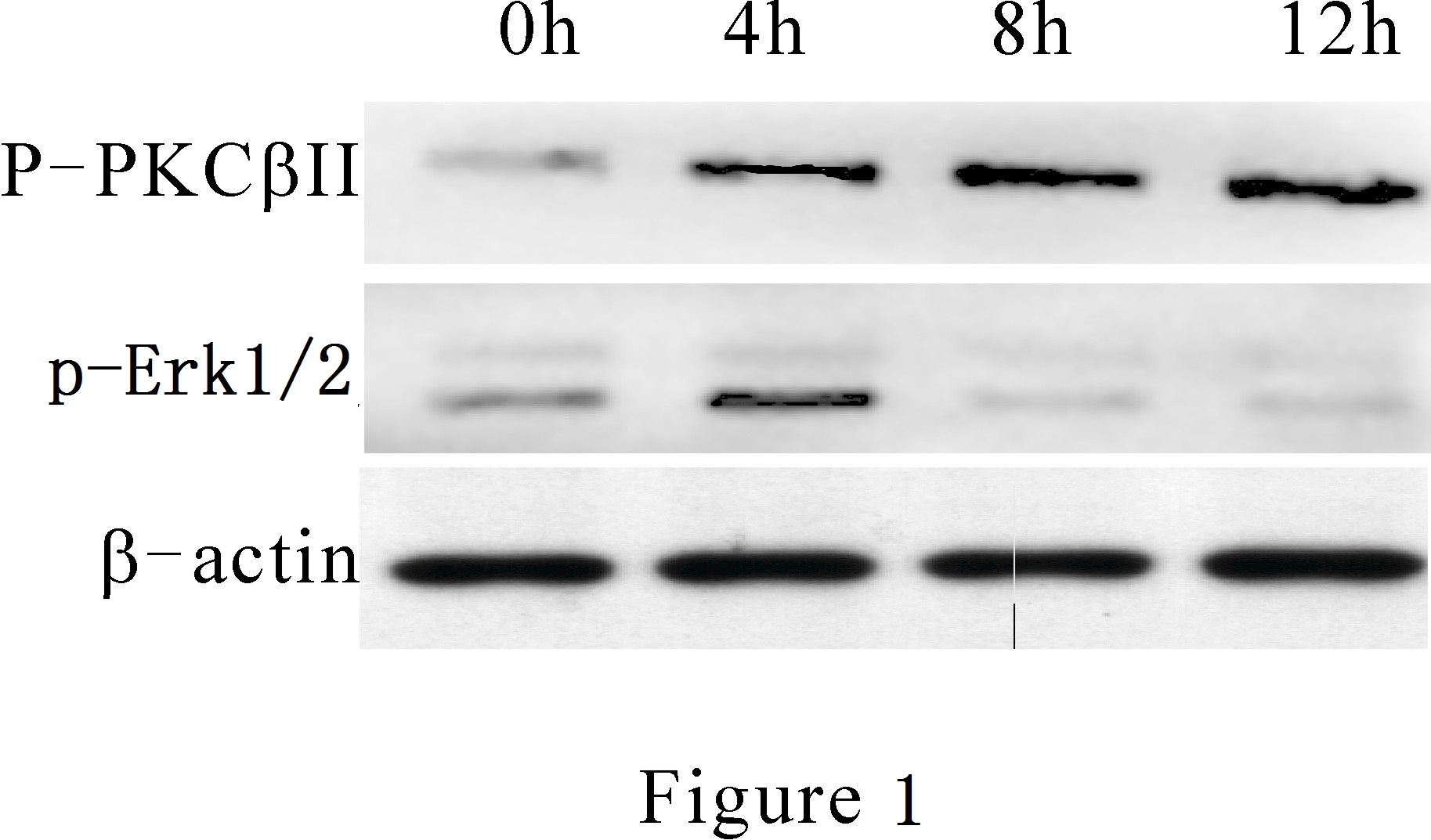

Supplement: Supplementary file 1 — Figure S1 HMGB1 activated the PKCβ/Erk1/2 in cardiac fibroblasts/myofibroblasts. Cardiac fibroblasts/myofibroblasts were treated by 100 ng/ml HMGB1. After 4, 8 and 12 hrs, cardiac fibroblasts/myofibroblasts were harvested; phosphorylated PKC β (p-PKC β) and phosphorylated Erk1/2 levels were assessed by western blot. Representative blots are shown above. Data were obtained from three independent experiments. [file jcmm0018-1740-SD1.jpg]
